# Exploration of dynamics in a complex person-centred intervention process based on health professionals’ perspectives

**DOI:** 10.1186/s12913-018-3218-3

**Published:** 2018-06-13

**Authors:** Febe Friberg, Catarina Wallengren, Cecilia Håkanson, Eva Carlsson, Frida Smith, Monica Pettersson, Elisabeth Kenne Sarenmalm, Richard Sawatzky, Joakim Öhlén

**Affiliations:** 1 Faculty of Health Sciences, University of Stavanger, 4036 Stavanger, Norway; 2 University of Gothenburg Centre for Person-Centered Care (GPCC), Gothenburg, Sweden; 3 Institute of Health and Care Sciences, Sahlgrenska Academy at the University of Gothenburg, P.O.Box 457, 40530 Gothenburg, Sweden; 4 Department of Nursing Science, Sophiahemmet University, P.O. Box 5605, 11486 Stockholm, Sweden; 5 Department of Surgery Sahlgrenska University Hospital/Östra, 416 85 Gothenburg, Sweden; 6 Chalmers University of Technology Division of Service Management and Logistics Department of Technology Management and Economics Chalmers University of Technology, Vasa hus 2, 412 96 Göteborg, Sweden; 7 The Vascular Department, Sahlgrenska University Hospital/Sahlgrenska, 416 85 Göteborg, Sweden; 8 Research and Development, Skaraborg Hospital, Skövde, Sweden; 9 School of Nursing, Trinity Western University, 7600 Glover Rd, Langley, BC V2Y 1Y1 Canada; 10 Centre for Health Evaluation and Outcome Sciences, Providence Health Care Research Institute, 588 – 1081 Burrard Street, St. Paul´s Hospital, Vancouver, BC V6Z 1Y6 Canada

**Keywords:** Complex intervention, Colorectal cancer surgery, Person-centred care, Preparedness, Professionals’ perspectives, Process evaluation

## Abstract

**Background:**

The assessment and evaluation of practical and sustainable development of health care has become a major focus of investigation in health services research. A key challenge for researchers as well as decision-makers in health care is to understand mechanisms influencing how complex interventions work and become embedded in practice, which is significant for both evaluation and later implementation. In this study, we explored nurses’ and surgeons’ perspectives on performing and participating in a complex multi-centre person-centred intervention process that aimed to support patients diagnosed with colorectal cancer to feel prepared for surgery, discharge and recovery.

**Method:**

Data consisted of retrospective interviews with 20 professionals after the intervention, supplemented with prospective conversational data and field notes from workshops and follow-up meetings (*n* = 51). The data were analysed to construct patterns in line with interpretive description.

**Results:**

Although the participants highly valued components of the intervention, the results reveal influencing mechanisms underlying the functioning of the intervention, including multiple objectives, unclear mandates and competing professional logics. The results also reveal variations in processing the intervention focused on differences in using and talking about intervention components.

**Conclusions:**

The study indicates there are significant areas of ambiguity in understanding how theory-based complex clinical interventions work and in how interventions are socially constructed and co-created by professionals’ experiences, assumptions about own professional practice, contextual conditions and the researchers’ intentions. This process evaluation reveals insights into reasons for success or failure and contextual aspects associated with variations in outcomes. Thus, there is a need for further interpretive inquiry, and not only descriptive studies, of the multifaceted characters of complex clinical interventions and how the intervention components are actually shaped in constantly shifting contexts.

## Background

The assessment and evaluation of practical and sustainable development of health care has become a major focus of investigation in health services research [1]. In line with this, complex clinical interventions are increasingly used and there is a growing body of literature regarding related methodologies [2–4]. The ultimate goal of complex interventions is to improve health care practices [5] that are often characterized as complex systems and therefore multifaceted with constantly shifting contexts [6]. Conceptualizing the practical workability of new treatments and behavioural approaches and assessing their potential for integration in healthcare settings are key issues for research. It is therefore important to evaluate how complex interventions are processed by professionals.

In this study, we investigated health professionals’ (nurses’ and surgeons’) perspectives on being part of an intervention process in a multi-centre intervention project based on a quasi-experimental design. In addition, we used participatory action research (PAR) procedures [7] for the development of one of the intervention components (the innovative PEM). The intervention aimed to support and help people diagnosed with colorectal cancer (CRC) to feel prepared for surgery, discharge and recovery by facilitating person-centred communication between patients and professionals. Such communication can be seen as an attempt to shift health professionals’ attitudes from seeing patients as passive recipients of care to seeing them as active partners of the health care team, with experience, context, capability and own resources [8]. The goal is to enable better care in a partnership between patients and professionals through dialogue and democracy in decision-making [9]. The motive for initiating this intervention partly came from the fact that two nurse managers at one of the hospitals contacted the researchers about the need to improve patient information in connection with discharge routines after colorectal cancer surgery. Thus, the intervention was requested and not imposed from one of the hospitals included, which may have importance for understanding the results.

In the next section, the development of the intervention will be described to make it easier for the reader to understand the bases for the current study. This is followed by a presentation of complex interventions and process evaluations.

### Intervention background

#### Surgery–recovery process for patients undergoing colorectal cancer surgery

In Sweden (including the study hospitals), the care process in CRC surgery and recovery follows national and international guidelines [10]. Treatment decisions were made in multidisciplinary conferences and all patients received at least one consultation pre-surgery, one before discharge, one after the surgery and one follow-up consultation with a surgeon after discharge. Pre-surgery patients had consultations with additional representatives of the multiprofessional CRC team, including a preoperative information consultation with a registered nurse (RN). The widely used multi-modal care pathway for the surgical process was applied by implementing the Enhanced Recovery After Surgery (ERAS) protocol. This protocol is designed to minimize the stress response associated with surgery and proven to be best evidence-based practice during pre- and postoperative care [10, 11]. The outcomes are improved recovery, fewer complications and a reduced hospital stay. Patients scheduled for ostomy surgery meet an enterostomal therapist pre-surgery and regularly following surgery. Results of tumour diagnosis assessment (pathological and anatomical diagnosis) are given to patients at a follow-up consultation with the surgeon usually within 4 weeks after surgery, and if adjuvant chemotherapy post-surgery is recommended, the patients are referred to an oncology department. According to national guidelines, at time of diagnosis all patients should be assigned a cancer nurse coordinator (in Swedish literally contact nurse) for support in relation to self-care, to navigate in the care process and to receive psychosocial support.

### Preparatory studies motivating the intervention development

In order to explore and understand current patient information practices in connection with CRC surgery, the intervention was developed on the basis of preparatory studies. Existing patient education materials (PEM) for CRC surgery were studied as related to patients’ preferences thereof [12] and existing discourses in the PEM [13], characteristics of pre-surgery CRC consultations [14] and physicians’ strategies to enable gastro-intestinal cancer patients’ understanding of bodily changes [15]. The results revealed an obvious need to develop new PEM of high quality in regard to suitability, readability, comprehensibility and content fitting patients’ preferences [12, 13]. The results of the preparatory studies also indicated a need for communication that takes patients’ narratives, perspectives and concerns into account [14–16].

### The intervention

**Fig. 1:**
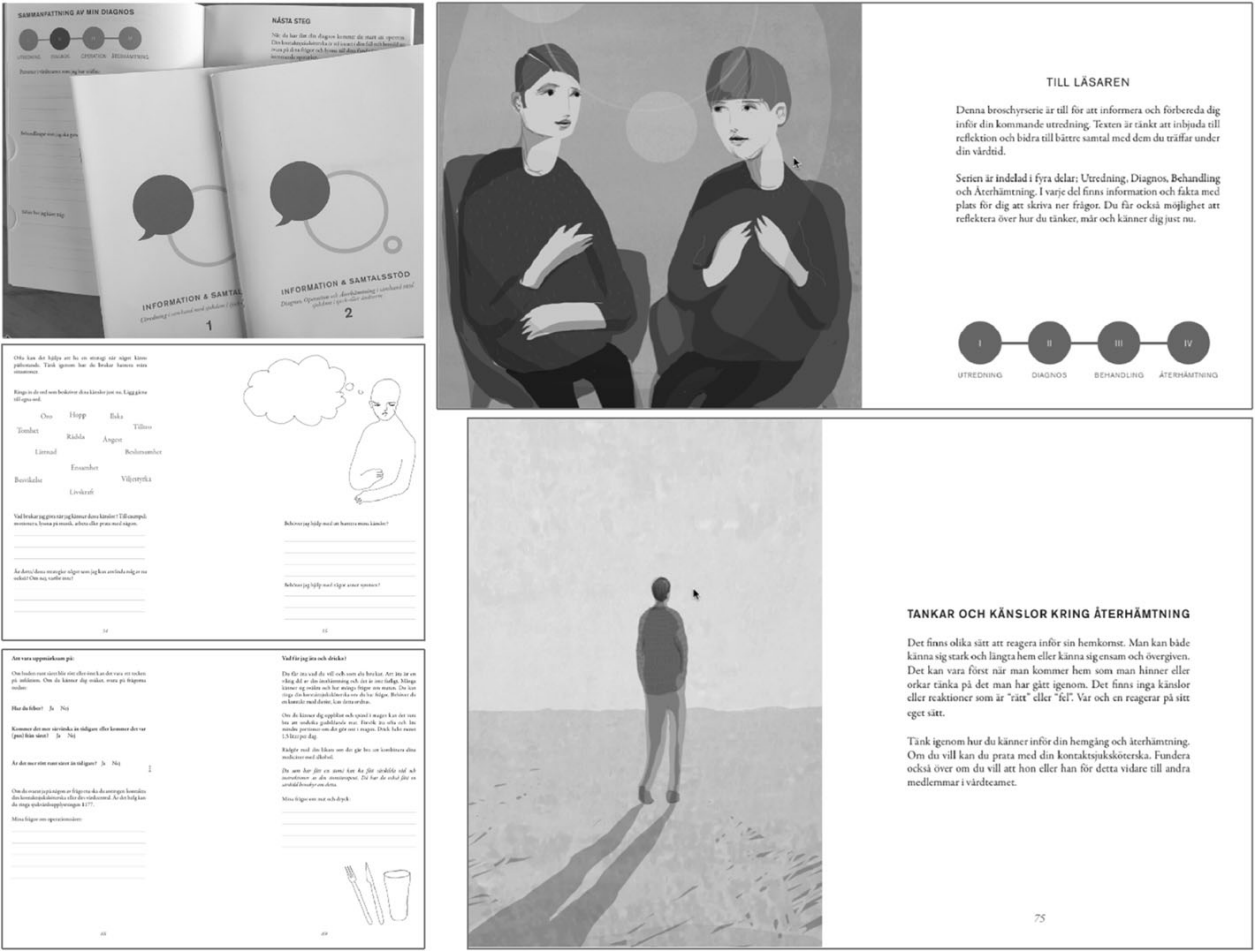
Examples from the PEM. Top left cover of the two brochures, top right, first spread in part 1“To the reader” with introduction to the PEM with four chapters with symbols used to support the reader in understanding the care process: examination, diagnosis, surgery and recovery (the symbols return in every chapter). Bottom left, two spreads with examples of how self-reflection is facilitated. The first allows the reader to circle words expressing their own emotions and write down strategies for dealing with struggling emotions. The second, in the left corner, has one page with questions to support self-care assessment of the wound after surgery and instructions regarding when and whom to contact for professional assessment and care. There is also a page with information about what to eat and drink, and both these pages have space for “My questions about the wound/food and drinks”. The bottom right page discusses “Thoughts and feelings around recovery”: “People react in different ways before coming home. You may feel strong and longing to come home or lonely and abandoned. Perhaps, once you’re home, you have time and strength to think about what you have gone through. There are no ‘right’ or ‘wrong’ feelings or thoughts. Everyone reacts in his or her own way. Think about how you feel about coming home and your recovery. If you like, you can talk to your cancer nurse coordinator. Consider whether you would like him or her to pass on your thoughts to other members of the health care team”. Illustration copyright: Helena Kjellgren

The intervention was based on two components: (i) the use of supportive interactive patient education material (PEM) and (ii) person-centred communication approaches through dialogues. Firstly, a new PEM was developed in cooperation with patients and professionals, using participatory action design (PAR) methodology [7, 17] Fig. 1.

**Fig. 2:**
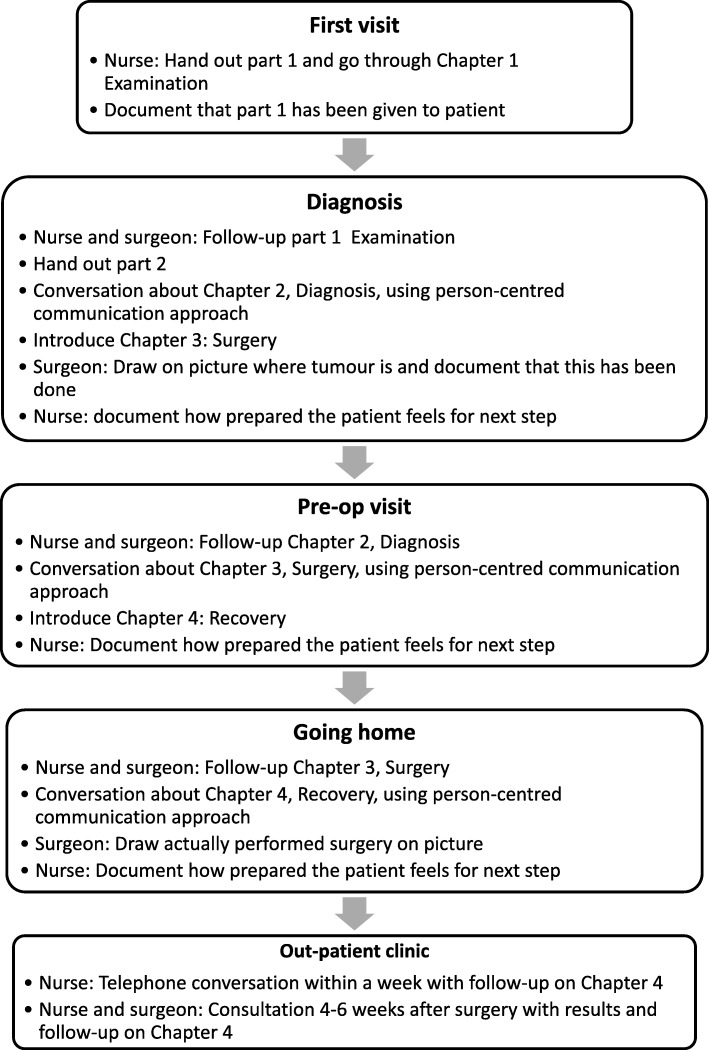
Overview of ways the PEM was to be used in the intervention

This was structured into chapters labelled as four phases of the care process: examination, diagnosis, surgery and recovery, and designed to serve three purposes: (i) To provide generic information in relation to the CRC surgery and recovery process of relevance on a group level (3rd person perspective; what is usually considered a PEM). This was based on best evidence-practice and current legislation, and with language and layout to promote high readability, suitability and comprehensibility, considering that the cancer diagnosis might affect patients’ health literacy. (ii) To enable opportunity for dialogue (2nd person perspective) primarily between patient and professionals (but also between patient and significant others). Here, both the patients and the professionals should be encouraged by the PEM to voice concerns and share their perspectives, and ultimately to form a partnership in which capacities, resources and needs can be discussed. (iii) To allow the patient to personally reflect (1st person perspective) on the generic information that has been processed in dialogue, and then for them to articulate and reflect on related assumptions, desires and behaviours, to assimilate the surgery and recovery situation. For details, see Smith [7] and examples from the PEM given in Fig. 1. The PEM was used in different consultations and in different ways and is displayed in Fig. 2.

**Fig. 3:**
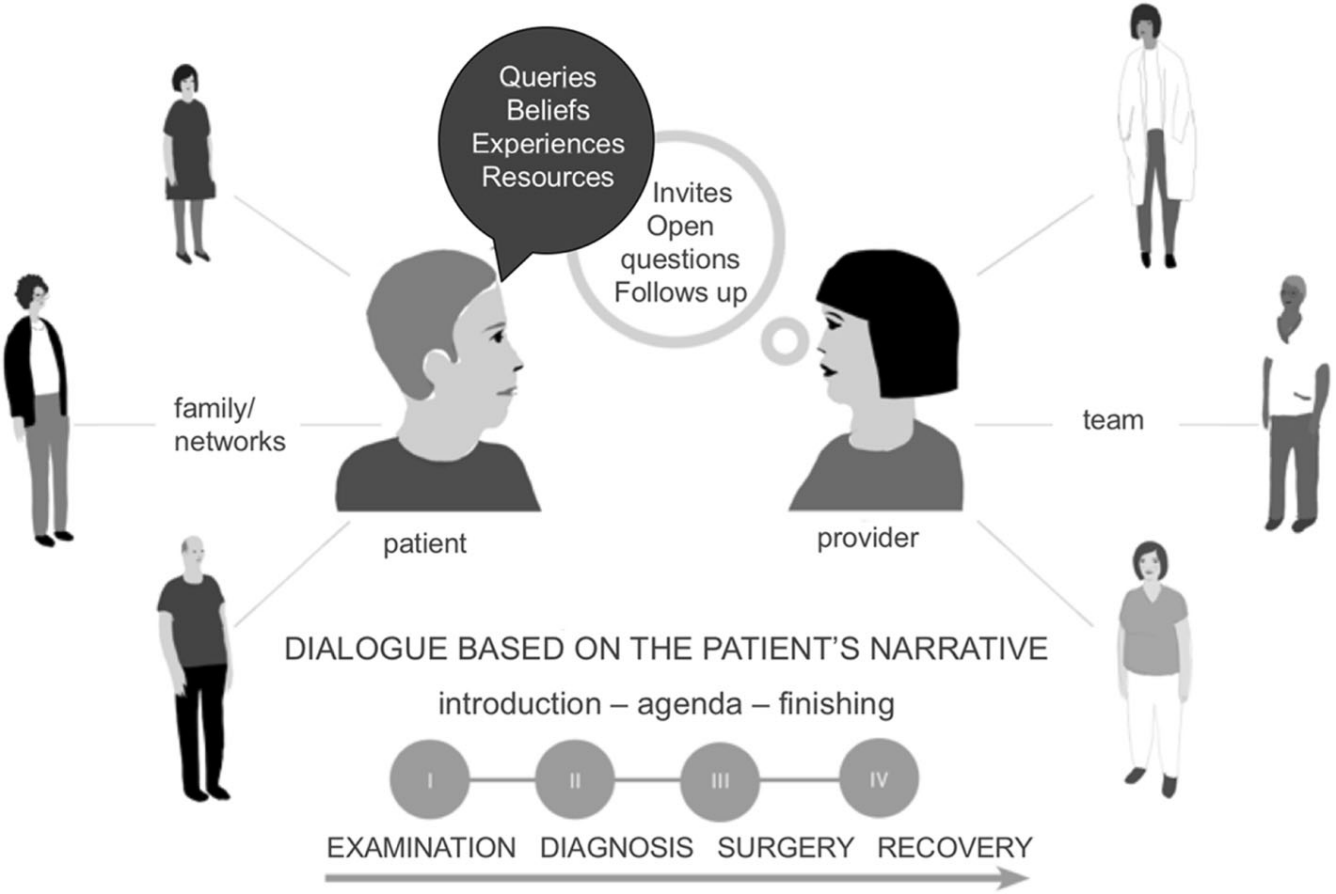
Illustration of the intervention component *person-centred communication in dialogues*; adapted to CRC surgery care. Illustration copyright: Helena Kjellgren

The new PEM consequently supported professionals in carrying out person-centred communication [8, 18] during consultations, including the following facilitating communication structures: (i) professionals guiding the patient through the four phases of the care process; (ii) explicitly communicating an introduction, agenda and closing of every consultation; (iii) inviting the patient to discuss and following up on what has been said, as well as posing open questions to invite the patient to enter a dialogue based on his/her story; and (iv) being sensitive to the patient’s questions, beliefs, experiences and resources Fig. 3.

This was contextualized in the patient’s family and social network and the professional’s team. Based on the dialogues, it was suggested that nurses should document aspects of how the patient was prepared for the surgery, the discharge and the recovery in the patients’ records. To introduce the intervention, all professionals assigned to the intervention wards at the three hospitals were invited to a two-hour workshop (in total 20 workshops and 251 professionals), which included a brief lecture on person-centredness and person-centred communication and an explanation of the two intervention components. Thus, objectives, theoretical bases, components and protocol were introduced. A video concretizing and illustrating the two components of the intervention (produced by the research team), and discussions and reflections were integrated throughout the intervention.

To secure intervention fidelity, facilitators (nurses and surgeons) were assigned locally at the three hospitals, with the intention of helping the other health professionals to work in accordance with the intervention protocol [19]. These facilitators were the primary contacts with the research team. During the ongoing intervention, workshops and regular follow-up meetings were held with facilitators (nurses) and representatives for cancer nurse coordinators at each hospital.

An introductory intervention kit for self-directed studies was developed for the professionals who did not take part in the introductory workshop. It also functioned as a supporting tool for all of the participants. The intervention has now been finalized and the outcome data is under analysis.

### Complex interventions and process evaluations

There is no absolute definition of a complex intervention. According to Craig et al. [3] complexity is determined by interacting components within the intervention and control groups, actions required by the individuals delivering or receiving the intervention, organizational levels or groups targeted by the intervention, variability of outcomes, and finally, how far tailoring of the intervention is permitted [3]. In this study, a change in approach among the health professionals from transfer of information to dialogue-directed communication was necessary. The complexity of the intervention relates to several interacting aspects: theoretical assumptions underlying person-centred communication and preparedness; procedures that professionals should adopt based on person-centred communication; involvement of different professionals; inter-professional collaboration, primarily among nurses and surgeons across different health care settings.

A key challenge for researchers, as well as decision-makers in health care, is to understand mechanisms influencing how complex interventions work and become embedded in practice, which is significant for both evaluation and later implementation [20–22]. Despite a growing body of knowledge about the importance of including process evaluations as a component in the development of clinical complex interventions [4], reports of process evaluations occur to a minor degree. Although there is substantial evidence, knowledge is still lacking on how complex interventions are best performed and implemented on a larger scale to contribute to sustainable development in health care. Several researchers [23–25] point to the need for process evaluations that consider the complexity of the “real world” and messy practical problems to a higher extent. Although it is important to study causal mechanisms [1, 21] contextual and pragmatic circumstances have to be considered [22, 25, 26]. Multi-method strategies, including interviews with stakeholders to grasp the complexity, are suggested [24, 27].

There is no consensus regarding what a process evaluation should include in detail and there are differences in how terms are used in evaluation frameworks. In this study, we specifically consider the theory base for the intervention (theoretical assumptions), the character of implementation components, how these are perceived by professionals, context and contextual factors, and mechanisms of impact [20, 23, 24] that operate in a particular context to produce an outcome [25]. Such aspects of process evaluation explore how the intervention is implemented and reveal insights into reasons for success or failure and contextual aspects associated with variations in outcomes [3]. These also enhance understanding of how an intervention could move from research to practice [1].

The aim of this study was to explore health professionals’ understanding and perspectives on performing and taking part in a person-centred information and communication intervention process with the goal of enhancing patients’ preparedness in connection with elective surgery for colorectal cancer. Consequently, the study intends to shed light on problems and possibilities related to processes of implementation, especially in gaining insights of importance for future implementation.

## Methods

### Design

A qualitative interpretive description design [28] was applied in the current study. The basis of interpretive description is enquiry into phenomena of interest to healthcare disciplines. As such, it recognizes that reality, i.e. in this study being in the midst of the intervention, is complex, socially constructed and intersubjective [28]. The enquiry was focused on obtaining experiential and practice-based knowledge from health professionals, with the ultimate goal of generating applicable knowledge about complex care interventions of relevance for clinicians, policy makers, and researchers. Thus, the process evaluation in this study was implemented by the qualitative methodology interpretive description.

### Settings and participants in reflective interviews

Three hospitals were selected for the intervention based on organizational and geographical differences: one university hospital, one regional hospital and one local non-profit hospital in Sweden. In this study, strategic sampling was the primary sampling principle in the selection of professionals from the three hospitals. They had all taken part in delivering the intervention and were assumed to have comprehensive experience of using it. Ten nurses and 10 surgeons from the three hospitals agreed to participate in retrospective reflective interviews after the intervention project was finalized. Number of years of working experience in CRC care ranged from 2 to 31 years among the surgeons. All except one of the surgeons were specialized in CRC care. For the nurses, the CRC experience ranged between 7 and 30 years. Seven of these were cancer nurse coordinators. All nurses had specialized nursing education and some more than one specialization.

### Data sources

A combination of strategies was used for the generation of data: reflective interviews, workshops and follow-up meetings. The primary data source was the retrospective reflective interviews, conducted in Swedish with nurses and surgeons. The workshops and follow-up meetings were considered complementary data sources and these were generated during the ongoing intervention.

### Reflective interviews

The reflective interviews, performed between January – March 2016 (when the intervention was finalized), were in conversation format and took place in quiet locations within the hospital settings. Four focus groups and four individual interviews were performed, depending on the participants’ working situations.

One interviewer led each session, apart from one of the focus groups, in which two interviewers participated. The participants were invited to talk about experiences related to the intervention. During the sessions, the interviewer highlighted and returned to mention or implicitly indicate aspects that were relevant to the study aim. Examples of questions that reiterated previously mentioned topics were: Can you please tell me how the intervention was introduced? How was it delivered? What was problematic? What went well? What are the potential benefits? Each session ended with the interviewer summarizing the topics raised during the interview, with the participants being invited to add comments and reflections. In this way, the first and latter parts of the interviews emphasized shared understanding, while the middle part explored potential issues and tensions. The focus groups lasted 35–60 min, including small talk, information, presentation rounds and so forth, whereas the individual interviews lasted 30–45 min.

### Workshops and follow-up meetings

Two types of workshops were performed during the ongoing intervention with six months between the events. The workshops included nurses who facilitated the study at the participating hospitals (see Table 1).

**Table 1:** Data generation

Data source	Time of data generation	Type of data	Participants
*Workshop (5 h)*	Six months into the intervention In total 1 workshop at one of the hospitals	Digital recordings/transcripts (*n* = 7)	Facilitators and cancer nurse coordinators participating in the project (*n* = 15)
*Workshop (3 h)*	The last three months of the intervention. In total 3 workshops at the different hospitals	Digital recordings/transcripts (*n* = 3)	Facilitators (nurses) at the different hospitals (*n* = 14)
*Follow-up meetings*	Continuously during the intervention In total, 9 meetings at the different hospitals	Notes from 6 meetings (*n* = 6)	Facilitators (nurses and surgeons) (*n* = 22)
*Focus groups* (four groups, A-D)	After the intervention	Digital recordings/transcripts (*n* = 4)	4 physicians (hospital I) 4 physicians (hospital II) 4 nurses (hospital I) 4 nurses (hospital II)
*Individual interviews*	After the intervention	Digital recordings/transcripts (*n* = 4)	1 physician (hospital III) 1 physician (hospital III) 1 nurse (hospital I) 1 nurse (hospital I)

In the first type of workshop (five hours), the main objective was to provide the nurses (*n* = 15) from the three hospitals with opportunities to share their experiences of being part of the intervention, and to offer support to facilitate the intervention. The workshops were framed by activities to promote a trustful climate in the group and a “World Cafe” approach to promote collaboration and dialogue, and facilitate sharing of experience-based knowledge [29]. The “Café” atmosphere [30] was created by laying a table with drinks and pastries, pens and paper and by providing a “host” (in our study, one researcher). The task of the “host” was to ensure that all the “guests” had their voices heard, that everything that was said was summarized and documented, and that new “guests” were brought into the topics discussed and ideas developed by previous guests.

Discussions in three groups (one researcher in each of the groups) were held for half an hour based on the following questions: How does the project work/function in your hospital? What are the current challenges? How can we support the progress of the project? Following this, the discussion continued in reorganized groups with one of the nurses and the researcher remaining as hosts while the other nurses moved about so that new groups were formed. After three rounds, the whole group gathered to share collective reflections. Digital audio recordings were made during all the group discussions.

The second type of workshop (3 h) was held at the three hospitals. The main objective of these workshops was to link back to the first workshop with reminders of the intervention objectives and components, and to provide opportunities to share experiences about the progression of the intervention. The workshops started with a researcher from the team presenting the current status of the project and a welcoming to promote a trustful climate in the group. The participants were invited to perform a SWOT analysis (a method with roots in the economics and business sector and used in connection to strategy work) of their unit’s strengths, weaknesses, opportunities, and threats [31] in relation to the intervention and its implementation. The results from the SWOT analyses were shared and discussed within the group at each hospital. Regular follow-up meetings with the facilitators were held in each hospital to support the ongoing intervention (see Table 1). During these meetings, experiences of the intervention were discussed. Ideas to help the professionals stick to the protocols and solve problems were developed in collaboration between the researchers and facilitators.

### Data analysis

**Fig. 4:**
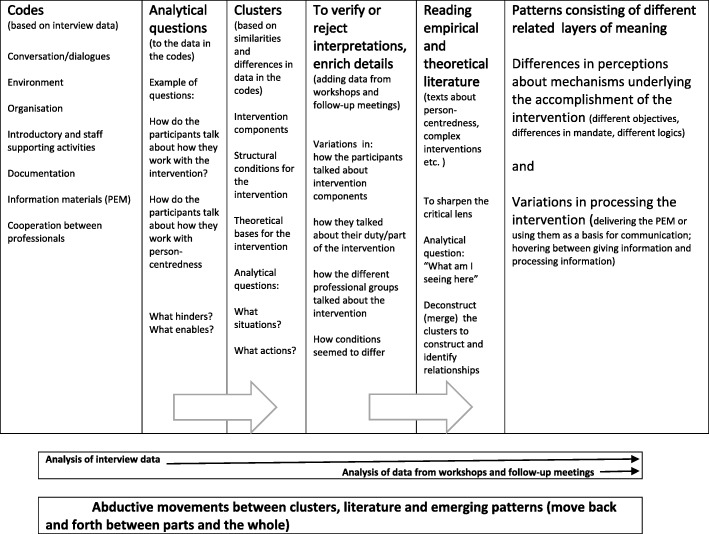
Analysis process

As seen in Fig. 4, the analysis first involved listening to the reflective interviews and reading the transcripts repeatedly to get an overall sense of the data before broadly coding and organizing it into clusters of text with related broader topics (e.g. matters regarding specific aspects of the intervention).

During this process we noted analytical questions such as: How do the professionals talk about how they work with the intervention process and person-centeredness? This was followed by comments and possible interpretations. These clusters of broad coded text were then further analysed and interpreted in the context of the overall understanding of the descriptions (i.e. in what situations, activities and so forth the descriptions were embedded). This was done by continuously moving back and forth between the clusters and the text as a whole. The additional data from workshops and follow-up meetings was then read in relation to the clusters from the analysis of the reflective interviews to check and enrich details, to verify or reject interpretations and to deepen the understanding of emerging patterns. Alvesson and Sköldberg [32] describe such movements between different analytical levels as abductive. This process involved moving back and forth between the data, the analytical notes and theoretical and empirical literature. Data on the different levels was compared with each other in order to identify more comprehensive meanings. Relevant empirical work by other researchers supported or rejected the interpretations, thereby sharpening them, while theoretical reasoning enhanced emerging understandings [28]. Examples of such sources are studies about complex interventions [2], process evaluation of complex interventions [11], person-centred communication [8], philosophy of the person [33] and professional logics [34]. In the final stages of the analysis process, a pattern consisting of two different layers of meaning was constructed; “Differences in perceptions about mechanisms underlying the accomplishment of the intervention” and “Variations in the processing of the intervention”.

As the idea of saturation is not applicable in interpretive description [28], we attained credibility through other principles typically applied in interpretive description, including: epistemological integrity (i.e. review of previous literature and thorough planning of the study design and methodology); representative credibility (i.e. striving to include different professionals on different occasions to get a variety of experiences, yet not claiming all the possible variations); and finally, analytic logic and interpretive authority, meaning that all researchers maintained a continuous exchange of reflective and critical reasoning about disclosure and interpretation of findings. Moreover, we discussed alternative interpretations, sometimes leading to re-analysis of the clusters of text with the purpose of confirming or rejecting the original interpretation. This was a transparent process, meaning that we shared ideas and thoughts about interpretations and agreed upon those before closure [28].

### Contextual conditions for the study

The participants in this study represented different professional groups and different socially and culturally shaped institutional settings, which presumably framed discussion about being part of the intervention. During the intervention period, certain organizational and administrative changes (change in leadership, new documentation systems, new research projects) occurred. Such institutional and other external factors were beyond the scope of the research team to influence. Another condition was related to the design of the intervention process. Despite holding introductory information and educational events to introduce the intervention (not part of the study analysis), and despite the workshops and follow-up meetings being described as informative and relevant, a need was articulated for additional follow-up meetings at regular time points over a longer period of time to ensure collective action and maintenance of the intervention. This indicated that the professionals might have needed more support, or support of a different kind, than was the case. Although the three intervention settings received the same introductory information, there seemed to be differences in how the intervention was embraced at each setting. Of significance were the social meeting places and thereby informative hubs, developed in connection with facilitating nurses’ work places. In summary, we assume that the participants’ utterances, which comprise the data in this study, were shaped by different circumstances and conditions.

## Results

In this study, we have unravelled the results we regard as most striking in understanding the functioning of the intervention. Patterns of two interrelated layers of meaning were constructed which each disclose significant practice-related details of the intervention process.

Influencing mechanisms underlying the accomplishment of the intervention will first be described, followed by variations in processing the intervention.

### Influencing mechanisms underlying the accomplishment of the intervention

Different influencing factors, here called mechanisms, were identified as related to the participants’ perceptions and actions. The term “mechanism” often implies a causal knowledge claim [11, 34] but in this study, we use it for culturally and structurally embedded factors through which the intervention activities (and outcomes) work. Although no specific questions related to mechanisms were elicited in the data, nearly all participants talked about or gave examples of incidents that could be related to this. Three suggested mechanisms will be described: differences in objectives, differences in mandates and differences in professional logics (Table 2).

#### Differences in perceptions about the intervention objectives

The overall (formal) intervention objectives were not explicitly discussed among the participants or in the interviews. Rather, diverse objectives were revealed in relation to improving the quality of patient information and patient information routines, communicating in such a way that no important information was missed, and considering patients’ perceptions, experiences and concerns in relation to the theoretical bases for person-centred communication. The most obvious objective, announced by most of the participants, was to *improve the quality of patient information* in connection with the pre- and post-surgical phases. This generic articulation was described by some of the participants as the primary goal of the intervention.

Another objective was related to the *improvement of the patient information routines* and the specific use of the PEM. This ensured that all patients received an appropriate level of relevant information. Another objective was to *communicate in such a way that no important information was missed*. The participants also mentioned the positive aspect of guaranteeing that all patients get the same information. Thus, various objectives were specifically related to the PEM.

In addition, and as a possible contrast, another objective was identified due to some participants also talking about considering the patients’ perceptions, experiences and concerns. Thus, beyond the accounts of the more generic objective (to improve the quality of information) some of the participants referred to *the theoretical bases for person-centred communication* as a reminder of how to act in the intervention.

Another element illustrating the theoretical bases for person-centred communication (introduced at the introductory information events for all professionals before the intervention) was related to difficulties in distinguishing the intervention component of person-centred communication from their ordinary professional approach to communication, which was also considered to be person-centred. The participants’ quotes illustrate this, for example: “I have always talked in this way” (nurse) or “I think I work in this way now” (surgeon).

Another example of an unclear relationship to the intervention objectives is illustrated by the following participant in a post-intervention interview:

Surgeon: “When I got to know that I was going to take part, I wondered “what is this intervention?”... and somehow I still don’t understand it. Just like, it’s this brochure and then there was a power point where it said you should document that you had drawn in the brochure…I hadn’t done that…I think…because I’m using my own words… I find it a little hard to use your words and…so I don’t really understand what the intervention is more than the brochure.”

Lack of clarity concerning the intervention objectives prompted this professional to act according to personal and experiential knowledge, thereby replicating previous habits (doing what they usually do). This can be seen as a reinterpretation of the formal and original intervention objectives (educated by the researchers). Thus, differences in perspectives and understanding of the intervention objectives can be interpreted as influencing participants’ choice of actions in the intervention.

#### Differences in perceptions about the mandate to work with the intervention

The intervention involved multiple professional groups and hospital settings. Although the participants’ utterances demonstrated differing proximities to the formal intervention objectives, they were nevertheless all supposed to take part in and deliver the intervention.

The group of facilitators were invited by the research team to have an explicit supporting role in the delivery of the intervention. They (most of them nurses) also seemed to have the most *well-defined mandate*. Many of them worked in outpatient clinics. The majority of the patients were invited to the project at the outpatient units and many had their first meeting with the surgeon there. The different intervention hubs gave opportunities for spontaneous intra- and inter-professional discussions and sharing of experiences, which nurses described as reassuring in terms of knowing what to do. In particular, the nurses expressed concerns related to differences in responsibility within their own professional group. In attempting to describe how the intervention followed the expected protocol or not, one facilitator said this about the way the ward nurses seemed to act:

Nurse: “No they aren’t interested...we still notice it now.” They’re not interested in this...it’s just another work task to complete or fill in. You know, we got comments like, “why do we need to have this?”

The lack of interest was described as frustrating, as the facilitating nurses were supposed to encourage the ward nurses and nurse managers to take ownership of implementing the intervention in the different surgical wards. Nevertheless, the facilitators acknowledged the relevance of the ward nurses’ hesitation to incorporate the content of the intervention into their already heavy workload. Consequently, in this case, *the mandate seemed to be ignored.* Although all the data demonstrated the facilitating nurses’ ambition to support the wards in using the PEM and in having person-centred discharge conversations, the question is how the ward nurses perceived their frames of action and mandate to act. Another dimension to attitudes towards the intervention mandate was illustrated in the following excerpts:

Surgeon 1: “I think there must be someone [a nurse] who says “now we’ll do it this way”– who reminds us. I can only see to myself…I do what I usually do and then something new comes along and you fall back into old habits again and then it’s like gone in a way… unfortunately, that’s the case.”

Surgeon 2: “I can’t manage to do it, I think...you fall back into old habits and stuff. Then you should have it repeated ...some type of information... I don’t know how to but.”

In this case, the responsibility to act in accordance with the intervention objectives seemed to be passed over to somebody else. The mandate to act appeared to be *redefined* as incorporating support and reminders from the nurses. If they were not reminded of the intervention components, the intervention was at risk of failing. Consequently, the variety of perceptions of the mandate among participants can be interpreted as influencing their choice of action in the intervention process.

#### Different types of professional logics

A third mechanism was related to different ways of organizing the intervention activities in parallel with the participants’ ordinary professional duties. The term “logic” is here understood as the rationality in an institutionalized working order which also relates to goals and means to reach that goal [34]. A knowledge-oriented professional logic and an administratively oriented professional logic will be described. The following remark made by one of the participants indicates the complexity in balancing a professional and participatory research agency: “This [the name of the project] is not just a research project but a way of working [integrated in ordinary daily work].”

*A knowledge-oriented professional logic* was identified in the data; more precisely, a medical professional and a nursing professional logic. The medical professional logic was most visible. Surgical departments specialising in colorectal cancer are by definition knowledge-intensive settings. By tradition, this also entails professional authority, autonomy and independence [34], which contribute to culturally shaping the intervention settings.

In regard to the intervention components, the surgeons stated that the PEM and drawings in the PEM served their own professional interest, as they usually explain the anatomy of the colon to patients by drawing on a piece of paper.

Surgeon 1: “Yes, I don’t feel involved in the specific delivery [of the intervention]. I’ve received information and then we’ve used it [the PEM] with patients... or the little that we actually do ...but introducing it [the PEM] is perhaps NN [name of a nurse] and the enterostomal therapist, I think”.

Surgeon 2: “...we use it [the PEM] to fill in chosen parts, for example, the drawing-sketch there - I think I’ve used that part the most.”

Being expected to communicate in a person-centred manner in accordance with the intervention protocol was described by the surgeons as unproblematic and self-evident, as they always tried to talk that way. While the surgeons’ logic was obvious, the nurses’ professional logic was less visible. Although some professional knowledge-oriented logic was demonstrated in the nurses’ conversations at follow-up meetings, it was not as pronounced and clear as the surgeons’ logic. In parallel with this, we identified an *administratively oriented professional nursing logic*, as indicated by the surgeons’ comments suggesting that the nurses should support them with reminders to increase the likelihood of following the intervention protocol.

Surgeon: “It [the intervention] is so easy to forget but on the other hand we have it in this [the PEM] if we do forget… and we nearly always have the cancer nurse  coordinator with us and they are so good…so that means…it is perhaps thanks to them that we don’t forget as often.”

Another indication of this hierarchically oriented administrative logic was connected to pre-surgery consultations. Naturally, the surgeons were part of the pre-surgery diagnosis consultations, but the nurses (in most cases) only joined such consultations as listeners. Although both surgeons and nurses acknowledged this as positive for care continuity, the surgeons’ utterances indicate a lack of detailed knowledge about what nurses say and how they talk to patients, as well as a lack of understanding of the nurses’ role in discussing surgical care and recovery with them. When the surgeon finishes his/her part of the consultation, the nurse takes over and provides detailed, pre-defined pre-surgery information while simultaneously trying to accomplish the intervention component of person-centred communication.

Nurse 1: “... when it comes to our conversation [with the patient in connection with the medical consultation] I still feel that you...because there is a need for cooperation between the surgeon and the nurse …and for quite a long time it was…it’s a visit to the surgeon [medical consultation] …but we [the nurses] are also there…and then we have to have our information and that isn’t actually planned in the schedule.”

Interviewer: You have to have your information?

Nurse 1: I mean the brochure [the PEM] and so...

Interviewer: Yes …yes

Nurse 2: “And then ...it’s like this that we do this [information, the PEM] in this [the surgeon’s consultation] too…”

This meant the intervention was delivered under time constraints, leaving the nurses with less freedom and limited autonomy due to their apparently being more accountable to the institutional and administrative system.

Although the different professional groups worked together, they seemed to work in parallel from different professional agencies, which indicates a lack of inter-professional logic.

### Variations in processing the intervention

The differences in perceptions about the intervention objectives and mandates, as well as the different professional logics, set the stage for further exploration of the intervention process. Two interrelated themes were constructed, indicating parts that were significant for the accomplishment of the intervention and revealing to which extent the intervention was successful or weak.

#### The dynamics of delivering the PEM or using them as a basis for communication

It was apparent that the act of delivering the PEM was one of the most discussed aspects of the intervention at the follow-up meetings with the facilitators (nurses). Three different aspects of delivery were identified based on the participants’ discussion. Some participants (nurses) talked about *the very act of delivering the PEM* as practically the most important part of the intervention.

Nurse 1: “Well... we didn’t go through it carefully at all... most often ... we use the sketch and draw on the picture.”

Interviewer: The picture of the intestines?

Nurse 1: Yes and then also, as you say, there’s been a lot of information ...so it’s been quite easy to give it [the information]

Nurse 2: No not difficult, no

Nurse 1: No

Nurse 2: And it’s been good information to give.

Nurse 3: “There’s all you need in it [the PEM]”

Besides simply delivering the PEM, some participants specifically talked about *the structure of the PEM and how the different parts and chapters could be used* by the patients during the whole surgical care period and the recovery afterwards. Most efforts to guide the patients in using the PEM were made in connection with enrolment day and, to some extent, at discharge. In between these days, the nurses on the surgical wards were expected to encourage the patients to look at the PEM upon returning from surgical treatment. The facilitating nurses commented that this was challenging, mainly because of heavy work load and the severity of the patients’ medical situation. The further away from the hubs (e.g. outpatient units and preoperative units) the nurses were working, the more distanced the intentions of the intervention seemed to be to them.

A third aspect of delivery concerned *reminding patients to use the PEM*, as indicated by utterances such as, “Did you bring the PEM?” or “Don’t forget to read the PEM.” All the “delivery” approaches for how to use the PEM seemed to be based on some kind of knowledge transfer ideas. Thus, according to many of the participants, the PEM were delivered for the patient’s self-study as a basis for enhancing their ability to ask relevant questions.

In contrast, data from follow-up meetings revealed detailed descriptions of how to use the *PEM as a basis for the patient conversations*. Efforts to listen attentively to explicit and implicit questions, concerns and experiences while referring to appropriate chapters or parts of the PEM were described. Moreover, the facilitating nurses specifically talked about the PEM as personal information guides for home use or as enablers of preparedness for post-surgery medical consultations and telephone contact with the nurses.

All the participants regarded the PEM as very useful in their daily work, irrespective of whether they simply delivered them or actively used and thereby processed them. “Fantastic tools” and “Great tools for us and great for the patients” were just two of the common comments that reflected this. In the reflective interviews with surgeons, the PEM were described as a good structure “particularly for the nurses” as an aid in their information work to enable preparedness. Some even mentioned that the PEM were so good, they could almost completely replace parts of the regular medical conversation. This was related to the fact that some patients do not ask questions or actively participate in conversations. For such patients, the PEM can serve as self-study information materials and encourage them to formulate questions or concerns to be addressed when appropriate. In fact, the PEM were described as containing nearly everything a patient needs to know to enable preparedness for surgery and recovery.

Nurse: “And that’s what I think is so good about this material... everything we inform them about ... it really is there. So you never need to feel...I feel relaxed today now when I go in because I know that if I forget something, the patient has got it anyhow…because it’s written down there.”

As such, the PEM can be seen as assuring that the patient receives the most evidence-based, and up-to-date factual knowledge. This was described by all as time-saving and something that enhanced a sense of security.

Surgeon 1: “It saves doing many other things, other talks/conversations”

Surgeon 2: Yes and I also feel that there’s a lot of information ...if you know what it says [in the PEM] you can say “go home and read it in peace and quiet ...then you know that it [the information] doesn’t just fly over their heads when you’re rambling on.”

The PEM also contributed to continuity of care, as the patients were continuously reminded to bring them and to read relevant sections in order to be prepared for the different surgical care phases.

Nurse: “In this [the PEM] you have this line...the time line or I try to emphasize that “now you’re here...now you’re on your way to this place and eventually you will get here ...” There’s a chapter for every step...this is the new way of talking”

Overall, the PEM were used in different ways. Besides being a learning resource for the patients, the PEM also served as educational tools for the professionals who acknowledged their patient-teaching responsibility. For other professionals, the PEM served as a collection of relevant evidence-based and patient-safe facts.

#### Hovering between communication as “giving information” and “processing information”

Diverse perspectives on “dialogical conversations” were identified in the reflective interviews. Some of the participants (nurses) described having a cognitive and mental *awareness that person-centred dialogues were a component* of the intervention and that they were supposed to have such a dialogue with the patients. In addition, they knew that the intervention protocol suggested conversations begin with an open question and take place at different time points whenever possible.

Despite knowing this, some commented that in clinical practice, dialogical communication is an illusion. These participants deliberately chose to deviate from the intervention objectives (person-centred communication in a dialogue format, starting with an open question) and only delivered *information in terms of one-way communication*. The reasons described were lack of time, lack of willingness to change communication style or a conviction that there was no need for anything other than the evidence-based PEM content.

Nurse: “There I need to say ...because we are so pressed for time at enrolment...we have half an hour in which to give the patient the chance to reflect and ask questions...there is nearly no space for that because we have no time. And then they [the professionals] want them to be included in different studies… other studies than this then …and they [the patients] have to get information about that and then their heads are crammed and we have to give them information too…what happens is we can’t just take their questions…we also have to have time to give them information about the care period and what happens during the time they spend with us.”

Interviewer: So time is a...

Nurse 1: Time is difficult

Nurse 2: “Yes it is”

Other participants (nurses, surgeons) talked about “dialogical conversation” as a state of hovering between dialogue and one-way communication. They described how they struggled to fulfil at least some aspects of the component “dialogical conversation” and mainly prioritized “information giving” but tried very hard to answer some of the patients’ questions verbally or by pointing out answers in the PEM.

There were also participants (nurses) who said that they had changed their way of thinking about person-centred communication, partly thanks to what they learned at the introductory information event.

Nurse: “But then you had to do it on a patient by patient basis... and then we worked through this with the enrolment too...how we should talk to the patient... so I mean this was not clear from the first enrolled patient… we did that”

Interviewer: So you worked with it as you went along?

Nurse: Yes we did

Nurse: “And we changed some of our work too...Before, we had both specific preoperative information and the study and so we separated the two a bit… one of us focused more on the dialogue and one more on the studies so that we could really focus on the dialogue and keep other distractions at bay.”

The participants also pointed to the fact that their ability to see patients as capable persons with resources had increased and that they no longer saw them as simply having needs assessments.

Nurse: “...and you try to identify the patient’s goals and expectations in a way we perhaps haven’t done so much before.”

As a consequence, the patient as a person became visible, prompting nurses to change from a task-oriented focus in the conversation to *focusing on the patients’ concerns, questions and perceptions –* “you try to turn it around a bit”. Such reflective standpoints seemed to increase the potential to develop dialogues once the information was processed.

Nurse: “Most often, the patient has been thinking about this before...and they have prepared themselves...they’ve read the book [the PEM] in advance and when they come, you ask how prepared they are...most of them have put food in the freezer.”

Although the data contains statements and descriptions of incidents which indicate some kind of patient preparedness, the term ‘preparedness’ was seldom used. On the one hand, preparedness was related to enabling patient self-management in terms of knowing and acting in line with the professionals’ suggestions, such as early post-operative mobilization. One participant (nurse) clarified this by saying “This also involves challenging the patients’ arguments or standpoints when necessary”. On the other hand, other expressions of preparedness seemed to be integrated in the participants’ descriptions of how they acted in taking on board the patients’ perceptions and concerns to increase understanding of what might come. One nurse said:

Nurse: “Sometimes I backtrack and ask how things were before they came here... then you get to know and I think that’s something I have learned...yes, to think a bit more person-centred.”

Although not explicitly verbalized, enhanced preparedness appeared to be the intention. Concurrently, even under time constraints, some participants acknowledged the need to consider the patient’s rationality and way of reasoning in order to achieve person- centredness.

Although most of the nurses in the reflective interviews described themselves as having become more and more conscious about the term ‘preparedness’, only a few documented it in the patient record for the simple reason that they did not know where to record it. Another aspect was that no appropriate keyword was found in the existing electronic record systems.

## Discussion

In this study, we have analysed health professionals’ perspectives on taking part in and delivering a complex clinical intervention. We were specifically interested in understanding their experiences to shed light on the intervention process. The results indicate areas of ambiguity that significantly impacted on understanding the functioning of the intervention. Multiple objectives, differences in perceived mandate to accomplish the intervention and unclear professional logics are to be viewed as circumstances that affected how the intervention was taken on board and implemented. This may have influenced fidelity in terms of what was planned to be implemented in relation to what was actually implemented [11].

The importance of well-articulated and communicated theoretical assumptions underlying an intervention is argued as important for the planning of the intervention and interpretation of the effects [3, 11]. Although person-centred communication and preparedness were introduced and discussed at workshops before and throughout the intervention period, the results indicate differences in how this was understood. The results also indicate an ambiguity as to how to enable and practice person-centred communication. Parallels can be drawn to the results of a concept mapping study about patient-centred care [35] where the importance of a comprehensive view of humanity and partnership, knowledge about and influence of the health system and management and also professional identity and professional development of staff were identified as requirements for patient centredness. If we return to our process evaluation, all this may have influenced what was delivered in the intervention i.e. “the dose” [10] of the intervention and the manner in which it was delivered to the patient.

It is of ultimate concern that intervention components make sense to the professionals [5, 36]. In our study, it was obvious that the PEM stimulated the professionals to commit and engage, which can be seen as a success factor. Some critical reflections can be made. In contrast to the PEM, person-centred communication with emphasis on dialoguing was the intervention component that the professionals were more reluctant to discuss, and if they did discuss it, they did so very differently. Dialogical communication was regarded as important, but also self-evident. Most of the professionals claimed they already spoke to patients this way even before the intervention; however, our preparatory studies did not show that to be the case [20, 22]. Such an attitude seemed to make it difficult for participants to distinguish the intervention component “dialogical communication” from current practice. In line with Murray et al. [5], we found this intervention component did not completely make sense to the professionals. Similar results were found by Burau et al. [12] in a study about drivers and challenges in implementation of health promotion in community mental health services. Even if the participants in that study found the intervention components (health promotion) meaningful, their engagement varied, pointing to the significance of developing a more bottom-up understanding of context. The importance of sense-making in connection to implementation is also argued by Klemsdal [37], who points to the significance of switching from defence mode to learning mode in connection with implementation of an intervention. An alternative interpretation was that the professionals in our study perceived that they had no time to work in any other way than their traditional habitual one.

Unfortunately, despite iterative workshops and project follow-up meetings with the facilitators, we did not come as far as we would have liked. This may indicate limitations in the design of the intervention or in the ways in which professionals, including the facilitators, were supported to make sense of the intervention [27]. The results suggest that more preparatory effort was needed to prompt the professionals’ practice-based reflections in relation to the theory-based intervention components. Consequently, neither sense-making, nor collective action in terms of inter-professional teamwork were sufficiently achieved [5]. This was revealed by the differences in professional logics [34]. Also, while the surgeons’ logic was obvious, the nurses’ professional logic was less visible, indicating a medical hegemony.

One critical reflection is that we could have made use of additional pre-intervention investigation as to how the intervention procedures would affect the different professional groups in their daily work. However, we do not know to what extent professionals could have anticipated the most relevant mechanisms that interplayed in the intervention. Murray et al. [5] point to the significance of highlighting the benefits of participating in an intervention before launching one. All the professionals in the intervention wards were invited and encouraged to participate by their managers. Yet the professionals suggested additional and iterative workshops to increase coherence and sense-making of the intervention. Even if we recognize the pre-studies as very valuable for trying to make the intervention practice relevant, we assume we allocated too many resources to that part of the intervention development. More time should have been devoted to pilot testing with a small group of patients to identify weak parts of the intervention. In addition, a more participatory oriented approach with closer collaboration between researchers and professionals would probably have been beneficial.

Collective action in relation to shared objectives is required for a successful implementation [1, 6]. In our intervention, the organizational changes impacting on the intervention wards (change in leadership and administrative systems, staff turnover, new ward) influenced the extent of participation and also how the professionals made sense of the intervention, with negative impact on following intervention protocol.

Our study included colorectal wards at three hospitals, and different professional groups and thereby issues of context-dependent social processes. Although we involved specific facilitators, all the professionals were supposed to follow the intervention protocol. We tried to change information and communication routines in whole settings, which are in themselves professional practices embedded in social and hierarchical traditions [38]. The results indicate that both individual and organizational levels need to be addressed. Context, in particular, is often underestimated as a determining factor for the outcome of a complex intervention [3]. This is because contextual factors shape and even re-construct complex interventions and cannot be considered separate from such interventions [6].

To understand the patterns identified in the analysis, we will highlight some factors that possibly framed the participants’ discussion. Firstly, the motive for initiating this intervention partly came from two nurse managers at one of the hospitals. Thus, the intervention can be regarded as having a practice-initiated clinical interest. Upon launching the project, these managers took a more distanced role, leaving facilitating nurses with the responsibility for the intervention (with support from the research team). One reflection is that the managers could have been more involved in the different parts of the intervention. During this time there were also changes in leadership of nurses and surgery departments at two of the intervention hospitals. This may have influenced the participants’ “ownership” of the intervention. Another factor was that the three hospitals differed in size and organization (university, regional and local non-profit hospital). One only offered elective surgery and clearly formulated value-based goals for care and treatment, which may have influenced routines for patient information and communication in different ways. Two hospitals were contacted for participation after the proposal was fully developed and thus decided to participate based on a more developed intervention idea. There were also differences in staffing, for example, a higher proportion of nurses and specialized nurses enabling rapid recovery processes after surgery (ERAS nurses) and specialized nurses employed at the outpatient clinics.

The constructionist assumptions underlying the whole project and the process evaluation in the current study, indicate that the participants all contributed interactively in shaping conditions for the latter. The outcome evaluation of the intervention (to be reported in another article) is based on a quasi-experimental design, thereby intervening in social practices embedded and integrated in different social contexts. We included whole settings with all professionals to carry out the intervention instead of assigning only a few specifically trained professionals as interventionists. In addition we used PAR procedures [7, 39] for the development of the innovative written interactive PEM. This means that individual and collective perspectives, standpoints and actions influenced what we studied. All this contributed to the complexity of the intervention and to the participants’ reflections on how the intervention was carried out.

## Conclusions

The study indicates areas of ambiguities that significantly impacted on understanding how theory-based complex clinical interventions work. Moreover, the results display how interventions are socially constructed and co-created by professionals’ experiences, assumptions about own professional practice, contextual conditions and the researchers’ intentions. This study contributes to the significance of considering differences in perceptions about mechanisms underlying the accomplishment of the intervention and variations in processing the intervention (see Table 2). We assume that these aspects are applicable to complex clinical interventions in many cases. Process evaluations of complex clinical interventions are clearly advocated in methodological literature, but there are fewer empirical reports of process evaluations, in particular compared to the increasing number of effect evaluations that are actually found. A special feature of this study was the interpretive approach to layers of meaning in professionals’ experience to understand the functioning of the intervention. Thus, there is a need for further interpretive enquiry, and not only descriptive studies, of the multifaceted characters of complex clinical interventions and how the intervention components are actually shaped in “constantly shifting contexts” (Cf. e.g. 6).

**Table 2:** Critical mechanisms in the intervention process

Multiple objectives	Unclear mandates	Unclear and competing professional logics
Differences in objectives (general objectives, such as increased quality of patient information *versus* enabling of patient preparedness by means of person-centered information and communication) influence the ways of viewing or working with the PEM (simply delivering the PEM *versus* using the PEM as a basis for person-centred communication)	Differences in perceived mandate (well-defined mandate for the facilitating nurses *versus* redefined unclear mandate for the ward nurses; large degree of freedom for the surgeons) influence ways of viewing communication (more likely to process information in dialogical manner *versus* simply giving/transferring information)	Different professional logics (knowledge-oriented professional logic, surgeon or nurse oriented; administratively oriented logic) influence what part of the intervention was put in focus and the level of engagement

In this study, we would like to point out the challenges in assuming that the professionals should adopt the theoretical assumptions and procedures underlying person-centred communication. We had a strong ambition, at the same time knowing that cultural and hierarchical structures of knowledge takes time to change. Without this practical influence, the highly commendable intentions for clinical intervention research are at risk of largely remaining as rhetoric “ticking the boxes”, where we know more about its intended ideals than actual functioning in practice. Knowledge pertaining to how interventions work and resonate in practice is also crucial for relevant implementation of complex interventions tested in more or less experimental research designs.

## Abbreviations

CRC: Colorectal Cancer
ERAS: Enhanced Recovery After Surgery
PAR: Participatory Action Research
PEM: Patient Education Materials
